# Benign Cutaneous Neoplasms with Syndromic Associations

**DOI:** 10.3390/dermatopathology12040034

**Published:** 2025-10-08

**Authors:** Sean Lider, Chanel Mandap, Pavandeep Gill

**Affiliations:** 1 Island Medical Program, University of British Columbia, Victoria, BC V8W 2Y2, Canada; 2 Department of Pathology and Laboratory Medicine, University of Calgary, Calgary, AB T2N 1N4, Canada; 3 Alberta Precision Laboratories, Diagnostic and Scientific Centre, 9-3535 Research Road NW, Calgary, AB T2L 2K8, Canada

**Keywords:** benign skin tumors, benign cutaneous neoplasms, genetic syndromes, hereditary conditions, familial association, dermatopathology

## Abstract

There are many benign skin neoplasms encountered in dermatopathology practice that can be associated with underlying genetic disorders. Although benign themselves, these lesions can offer insight into the potential for development of internal malignancies in patients with these hereditary syndromes. An astute dermatopathologist will recognize clues that suggest a syndromic association of these lesions, such as the presence of multiple lesions, distinct histologic growth patterns, and the results of ancillary immunohistochemical testing. The dermatopathologist can then guide the referring clinician to obtain additional clinical and family history and, if appropriate, pursue further screening and genetic testing. This review article will provide an overview of the clinical and histologic features associated with select common and uncommon benign skin neoplasms with syndromic associations.

## 1. Introduction

Benign skin tumors are among the most frequently encountered lesions in dermatologic practice [1]. While they often arise sporadically and may be regarded as clinically insignificant, there are some instances in which the presence of these lesions may serve as important cutaneous indicators of underlying genetic syndromes, some of which are associated with an elevated risk of internal malignancy or other systemic complications [2]. In many cases, the skin provides the first manifestation of these conditions, with these benign skin neoplasms sometimes appearing years before internal disease becomes clinically apparent. Using cutaneous findings to identify an underlying hereditary condition presents a critical opportunity for early recognition and intervention in these genetic disorders [2,3].

This review highlights the clinical and histopathological features of select benign cutaneous neoplasms of adnexal, mesenchymal, and melanocytic origin with the aims of helping dermatopathologists become more aware of the possible syndromic associations of these lesions and recognize the clinical and histological clues that may prompt further recommendations to the referring provider to consider an underlying genetic syndrome in a patient.

## 2. Hair Follicle Tumors

### 2.1. Fibrofolliculoma/Trichodiscoma

**Figure 1:**
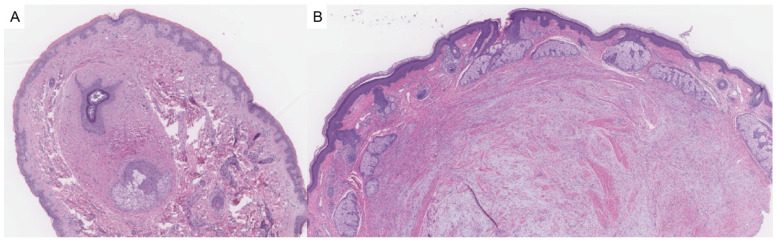
(**A**) Fibrofolliculomas are characterized by distorted or dilated central hair follicles embedded in a fibromucinous stroma (hematoxylin and eosin, 2×). (**B**) Trichodiscomas demonstrate a prominent fibromucinous stromal component with peripheral sebaceous lobules (hematoxylin and eosin, 2×).

Fibrofolliculomas and trichodiscomas are benign follicular hamartomas that may occur sporadically or, more notably, in association with Birt–Hogg–Dubé syndrome (OMIM# 135150). Clinically, these lesions present as small, dome-shaped, white-to-skin-colored papules most commonly distributed on the face, neck, and upper torso. In patients with BHD, they typically appear after puberty and may number in the dozens or even hundreds, sometimes coalescing into plaques [4]. Histologically, fibrofolliculomas are characterized by distorted or dilated central hair follicles embedded in a fibromucinous stroma with thin, anastomosing epithelial strands radiating from the follicular wall (Figure 1A). Trichodiscomas exhibit a more prominent stromal component and may include peripheral sebaceous lobules (Figure 1B). Both lesions are now considered histologic variants of the same process [5]. Immunohistochemical studies demonstrate diffuse expression of CD34 and scattered expression of factor XIIIa in the stromal component, with nestin and c-kit occasionally marking spindle-shaped stromal cells. The follicular epithelium may show aberrant CK15 expression. These findings support a shared origin in the differentiation of hair follicle bulge stem cells [5,6]. Markers of neural differentiation, such as S100, are negative in the stromal component [6].

Fibrofolliculomas and trichodiscomas are considered hallmark cutaneous findings of Birt–Hogg–Dubé syndrome, an autosomal dominant disorder caused by pathogenic variants in the *FLCN* gene on chromosome 17p11.2 [4]. BHD is associated with a spectrum of internal tumors, most notably renal neoplasms, including hybrid oncocytic tumors (most frequent), chromophobe renal cell carcinoma, renal oncocytomas, and less commonly, clear cell renal carcinoma [4,7]. Pulmonary manifestations are also common and include bilateral basilar lung cysts and recurrent spontaneous pneumothoraces [6]. While acrochordons may also be observed, they are not specific to BHD given their prevalence in the general population. Recognition of multiple fibrofolliculomas or trichodiscomas, especially in patients with a suggestive personal or family history, should prompt consideration of BHD and initiate appropriate genetic testing and systemic screening.

### 2.2. Tricholemmoma

**Figure 2:**
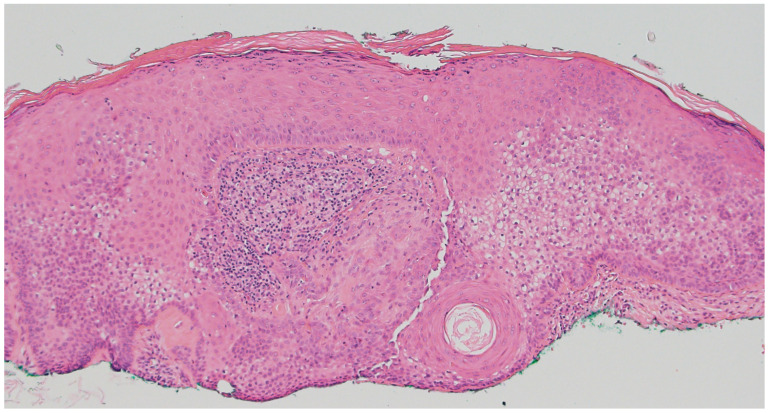
Tricholemmomas are superficial well-circumscribed lobular proliferations composed of pale or clear glycogen-rich keratinocytes with peripheral palisading and a surrounding thick basement membrane (hematoxylin and eosin, 4×).

Tricholemmoma is a benign follicular neoplasm that arises from the outer root sheath of the hair follicle and most commonly presents as a small, smooth, skin-colored or slightly pigmented papule on the face, particularly around the nose and upper lip [8]. These lesions are often solitary, but the presence of multiple facial tricholemmomas is a hallmark of Cowden syndrome and should prompt consideration of a syndromic association [9]. Histologically, tricholemmomas are well-circumscribed lobular proliferations composed of pale or clear glycogen-rich keratinocytes with peripheral palisading and are surrounded by a thickened basement membrane, mimicking the vitreous layer of the outer root sheath (Figure 2) [8]. Desmoplastic tricholemmoma demonstrates epithelial cords embedded in a dense sclerotic stroma, sometimes resembling an invasive carcinoma, such as basal cell carcinoma [10]. Periodic acid–Schiff (PAS) positivity, which is diastase-labile, highlights the glycogen content. Immunohistochemically, tricholemmomas typically express CD34 in diffuse or peripheral patterns, as well as CK17 and calretinin, supporting their outer root sheath origin [11]. A lack of expression for BerEP4 can help differentiate these lesions from basal cell carcinoma [12].

The presence of multiple tricholemmomas is strongly associated with PTEN hamartoma tumor syndrome (PHTS), a spectrum of autosomal dominant conditions caused by germline mutations in the tumor suppressor gene *PTEN* located on chromosome 10q23.31 [9,13]. PHTS includes Cowden syndrome (OMIM #158350), Bannayan–Riley–Ruvalcaba syndrome (OMIM #158350), Proteus syndrome (OMIM #176920), and Proteus-like syndrome. Among these, Cowden syndrome is the most well-defined and is associated with a significantly increased lifetime risk of malignancies including breast cancer (25–50%), follicular thyroid cancer (up to 10%), and endometrial cancer (5–10%), as well as possible increased risks of renal cell carcinoma, melanoma, and gliomas [9]. Other clinical features may include macrocephaly, mucocutaneous papillomas, acral keratoses, oral cobblestoning, multinodular goiter, and lipomas [9]. The dermatologic manifestations often precede the development of internal malignancies and serve as valuable diagnostic clues. Loss of PTEN protein expression on immunohistochemistry may be observed in these lesions and, if present, can support the diagnosis of PHTS, although its absence does not rule it out [14,15]. In a clinical context suggestive of PHTS, recognition of multiple tricholemmomas should prompt referral for genetic counseling and *PTEN* mutation testing, as early diagnosis enables appropriate cancer screening and risk reduction strategies.

### 2.3. Pilomatricoma

**Figure 3:**
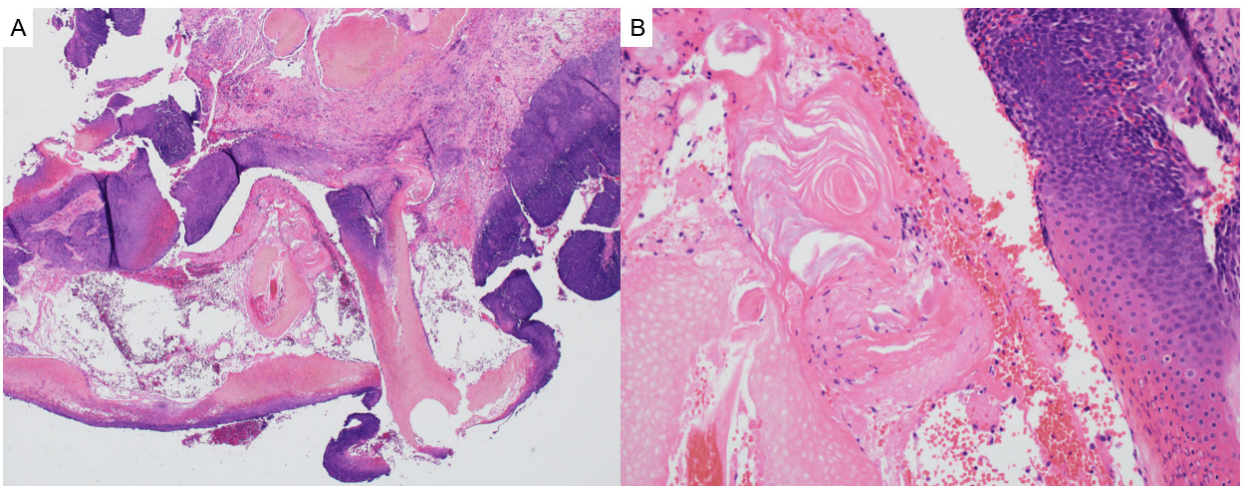
(**A**) Pilomatricoma may demonstrate rupture and are characterized as dermal or subcuticular tumors (hematoxylin and eosin, 2×) (**B**) composed of peripheral basaloid cells that transition abruptly into central anucleate eosinophilic shadow cells (hematoxylin and eosin, 20×).

Pilomatricoma, also known as calcifying epithelioma of Malherbe, is a benign adnexal tumor derived from immature hair matrix cells. It typically presents in children and young adults as a solitary, firm, slow-growing, subcutaneous nodule with overlying skin that may appear skin-toned, reddish, or bluish [16]. Lesions are most often located on the head, neck, and upper limbs, and commonly measure under 3 cm in diameter [16]. A useful clinical clue is the “tent sign”, where stretching the skin over the lesion reveals angular protrusions due to underlying calcification [17]. Histologically, pilomatricomas are sharply circumscribed, lobulated tumors of the dermis or subcutis, composed of peripheral basaloid cells that transition abruptly into central anucleate eosinophilic shadow (ghost) cells (Figure 3). Dystrophic calcification and foreign-body giant cell reaction are common features [18,19]. Immunohistochemically, nuclear and cytoplasmic accumulation of β-catenin is frequently observed in basaloid cells, consistent with dysregulated Wnt signaling [20]. This can result from activating mutations in *CTNNB1* or from upstream disruption of APC regulation in syndromic cases [19].

The presence of multiple pilomatricomas, especially six or more, warrants evaluation for syndromic associations, particularly Gardner syndrome, a subtype of familial adenomatous polyposis (FAP; OMIM #175100). Gardner syndrome is an autosomal dominant disorder caused by germline mutations in the *APC* gene on chromosome 5q21–q22 and is associated with colorectal adenomatous polyps (nearly 100% lifetime cancer risk) and extracolonic features such as osteomas, desmoid tumors, epidermoid cysts, supernumerary teeth, and retinal pigment epithelium hypertrophy [21,22]. Pilomatricomas in this context may occur as multiple independent lesions or as pilomatricoma-like changes within epidermoid cysts, showing histologic features such as shadow cells and basaloid matrical cells and nuclear β-catenin positivity [23,24]. Multiple familial pilomatricomas have been reported as early dermatologic signs of Gardner syndrome, and their presence, particularly in conjunction with a relevant family history, should prompt genetic counseling, colonoscopic screening, and *APC* gene testing [21].

### 2.4. Trichoepithelioma

Multiple trichoepitheliomas may be found in Brooke–Spiegler Syndrome (OMIM #605041) and multiple familial trichoepitheliomas (OMIM #601606). These autosomal dominant disorders are characterized by germline inactivating mutations in the tumor suppressor gene *CYLD*, located on chromosome 16q12-13. These syndromes are phenotypic variations in CYLD cutaneous syndrome.

Trichoepitheliomas present as skin-colored, non-ulcerated papules or nodules on the face, most commonly on the nose and nasolabial folds [25]. Syndrome associated tumors typically appear during puberty and progressively accumulate during adulthood. These tumors can number in the hundreds, with the potential to cause disfigurement [25,26]. Visual impairment and hearing loss can result from extensive involvement of the eyelids and external auditory canal [27].

**Figure 4:**
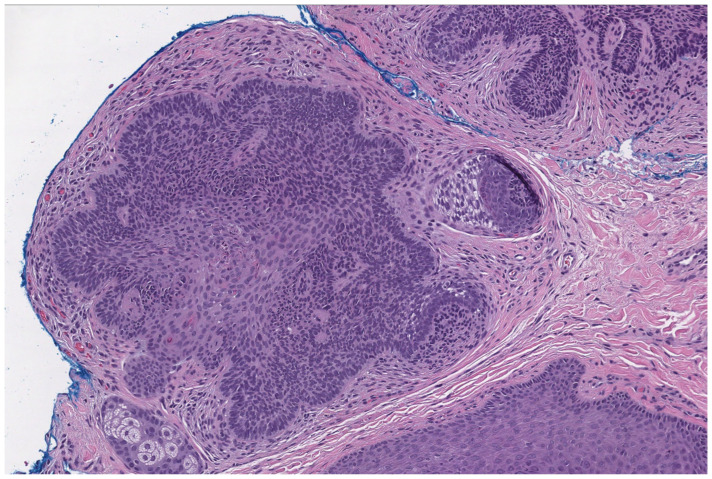
Trichoepitheliomas demonstrate nests of bland basaloid cells with peripheral palisading, associated papillary mesenchymal bodies, and a fibrous stroma (hematoxylin and eosin, 10×).

Histological examination of trichoepitheliomas demonstrates nests of bland basaloid cells with peripheral palisading, associated papillary mesenchymal bodies and horn cysts, and a fibrous stroma (Figure 4). Desmoplastic trichoepitheliomas are characterized by a prominent desmoplastic stroma surrounding small cords and islands of basaloid cells. Histologically, these lesions may mimic basal cell carcinoma [28]. The presence of numerous CK20-positive Merkel cells favors a diagnosis of trichoepithelioma [29]. BerEP4 is not helpful in distinguishing these lesions from basal cell carcinoma [30].

Brooke–Spiegler Syndrome is associated with the presence of multiple cylindromas, spiradenomas, spiradenocylindromas, and trichoepitheliomas [26]. Multiple familial trichoepitheliomas is associated with numerous trichoepitheliomas only. Malignant transformation of pre-existing skin neoplasms may develop in 5–10% of these patients [27]. *CYLD* cutaneous syndrome can also be associated with salivary gland tumors and rarely pulmonary and mammary cylindromas [27,31]. A *CYLD* cutaneous syndrome should be suspected in patients with multiple trichoepitheliomas with or without other skin adnexal neoplasms from a young age and with a family history of similar multiple tumors. Genetic testing can help facilitate early diagnosis and surveillance.

### 2.5. Basaloid Follicular Hamartoma

Basaloid follicular hamartomas may arise sporadically or be associated with nevoid basal cell carcinoma syndrome (NBCC)/Gorlin syndrome (OMIM #109400). This is an autosomal dominant disorder caused by germline mutations of the Sonic Hedgehog (SHH) pathway. Inactivation of genes, particularly *PTCH1* (chromosome 9q22.32) or *SUFU* (chromosome 10q24.31), leads to activation of the SHH pathway. This syndrome is characterized by an early age of onset of multiple basal cell carcinomas (the most common neoplasm in this syndrome), odontogenic keratocysts of the jaw, palmar or plantar pits, skeletal abnormalities, lamellar calcification of the falx cerebri, and characteristic coarse facial features (e.g., frontal bossing and macrocephaly) [32]. *SUFU* mutations are less prevalent and are associated with an increased risk of early-onset medulloblastoma [32]. Basaloid follicular hamartomas may represent up to 24% of skin tumors in NBCC [32]. Meningiomas, craniopharyngiomas, cardiac fibromas, and bilateral calcified ovarian fibromas may also be found in this syndrome [33]. Multiple basaloid follicular hamartomas in association with hypotrichosis may also develop in generalized basaloid follicular hamartoma syndrome (OMIM #605827), an autosomal dominant disorder of unknown molecular basis [33].

**Figure 5:**
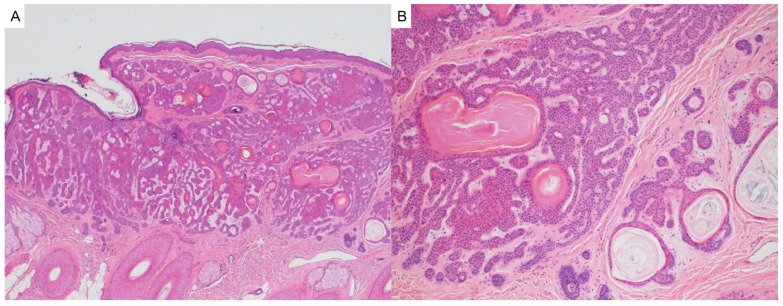
(**A**) Basaloid follicular hamartomas are symmetrical, well-circumscribed, superficial neoplasms (hematoxylin and eosin, 4×) (**B**) composed of radiating anastomosing cords and strands of basaloid and squamoid cells with associated horn cysts in a loose fibrous stroma (hematoxylin and eosin, 10×).

Clinically, basaloid follicular hamartomas are small, stable, slightly raised, skin-colored lesions on the face, scalp, and upper trunk [34,35]. On histology, basaloid follicular hamartomas may mimic infundibulocystic basal cell carcinoma and are characterized as symmetrical, well-circumscribed, superficial perifollicular neoplasms composed of radiating anastomosing cords and strands of basaloid and squamoid cells with associated horn cysts in a loose fibrous stroma (Figure 5). They demonstrate stromal-dermal clefting (but not tumor-stroma clefting), bland cytology, minimal mitotic activity, minimal apoptosis, and lack the peripheral palisading and inflammation that may be associated with basal cell carcinoma [32,34]. Immunohistochemistry can be helpful in distinguishing these lesions from basal cell carcinoma. Basaloid follicular hamartomas demonstrate the presence of CK20 positive Merkel cells (usually not present in basal cell carcinomas), focal EpCAM expression (diffuse expression in basal cell carcinoma), low Ki67 proliferative index (higher in basal cell carcinoma), and wild-type p53 expression (can be overexpressed in basal cell carcinoma) [32,35,36,37]. It is suggested that basaloid follicular hamartomas may be closely related or precursors to basal cell carcinoma [32].

Given the potential syndromic association and the risk for development of other associated lesions, the presence of multiple basaloid follicular hamartomas should prompt consideration for genetic evaluation.

## 3. Sebaceous Tumors

### 3.1. Sebaceous Adenoma/Sebaceous Epithelioma

Sebaceous adenomas and sebaceous epitheliomas are cutaneous adnexal tumors that can serve as clinical hallmarks of Muir–Torre syndrome (MTS), a subtype of Lynch syndrome [38]. Clinically, these neoplasms appear as solitary or multiple, small, yellow-to-pink papules or nodules, most commonly found on the face, scalp, and trunk [39].

**Figure 6:**
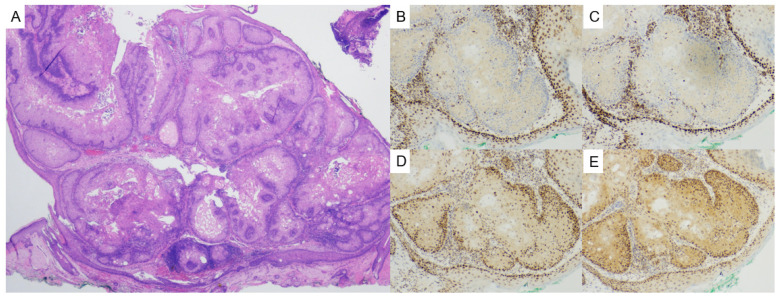
(**A**) Sebaceous adenomas are characterized as well-circumscribed, multi-lobular proliferations with multiple epidermal attachments and are composed of an admixture of mature sebocytes and basaloid cells with more than 50% of tumor being composed of mature sebocytes (hematoxylin and eosin, 2×). Evaluation of mismatch repair proteins in this sebaceous adenoma shows (**B**) loss of nuclear MSH2 (MSH2, 10×), (**C**) loss of nuclear MSH6 (MSH6, 10×), (**D**) retained nuclear expression of MLH1 (MLH1, 10×), and (**E**) retained nuclear expression of PMS2 (PMS2, 10×).

**Figure 7:**
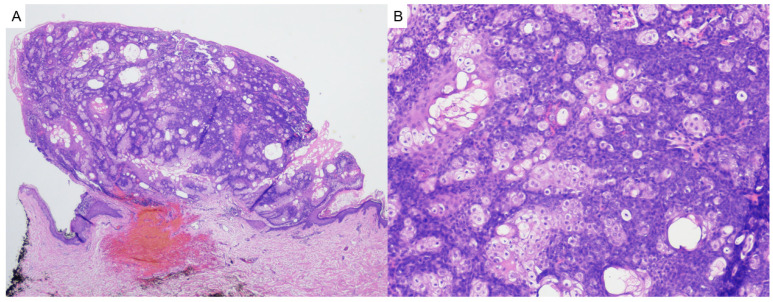
(**A**) Sebaceomas are characterized as well-circumscribed, multi-lobular proliferations with multiple epidermal attachments (hematoxylin and eosin, 2×) and (**B**) are composed of an admixture of mature sebocytes and basaloid cells with more than 50% of tumor being composed of basaloid cells (hematoxylin and eosin, 10×).

Histologically, sebaceous adenomas show well-circumscribed lobules with a predominant population (i.e., more than 50% of the tumor) of central mature sebocytes with a less predominant population of basaloid cells peripherally (Figure 6). Sebaceous epitheliomas (sebaceomas) are histologically similar but contain a higher proportion of basaloid cells (i.e., more than 50% of the tumor) (Figure 7) [38]. Sebaceous neoplasms in MTS may exhibit cystic or folliculocentric architecture [40]. Immunohistochemically, evaluation of DNA mismatch repair (MMR) proteins MLH1, MSH2, MSH6, and PMS2 is critical for identifying MTS-associated lesions. Loss of nuclear expression on immunohistochemistry for one or more of these proteins in tumor cells suggests mismatch repair deficiency and should prompt further investigation. Currently, a consensus screening strategy for MMR proteins in sebaceous tumors is not available [41]. The most recent American Society of Dermatopathology appropriate use criteria suggest a targeted screening approach to MMR immunohistochemistry, with a four antibody panel being appropriate in patients with multiple sebaceous tumors regardless of patient age, keratoacanthoma with sebaceous differentiation regardless of patient age, cystic sebaceous tumor regardless of patient age, MTS-associated neoplasm and/or visceral malignancy regardless of patient age, and sebaceous tumors arising in non-head and neck locations in individuals ≤60 years of age [42]. While immunohistochemistry is widely used, it does have a high false positive rate and low specificity [42], and germline sequencing remains the gold standard for diagnosing hereditary MTS [39].

Muir–Torre syndrome (OMIM# 158320) is an autosomal dominant condition caused by germline mutations in *MSH2* (accounts for ~90% of cases), *MLH1*, *MSH6*, and *PMS2*, all of which are components of the mismatch repair pathway [43,44]. Sebaceous neoplasms can precede, coincide with, or follow the diagnosis of internal malignancy, most commonly colorectal carcinoma, followed by genitourinary, endometrial, gastric, pancreatic, and breast cancers [45]. The presence of even a single sebaceous neoplasm—especially sebaceous adenoma, epithelioma, or carcinoma—warrants evaluation for underlying malignancy, particularly in patients with personal or family histories of Lynch-related cancers [40]. Keratoacanthomas have also been associated with MTS and may occur in combination with sebaceous tumors [46].

Detection of MMR deficiency in a sebaceous tumor, particularly with concurrent or personal/family history of visceral malignancy, should prompt genetic counseling, colonoscopy, and *MMR* germline testing.

### 3.2. Steatocystoma

**Figure 8:**
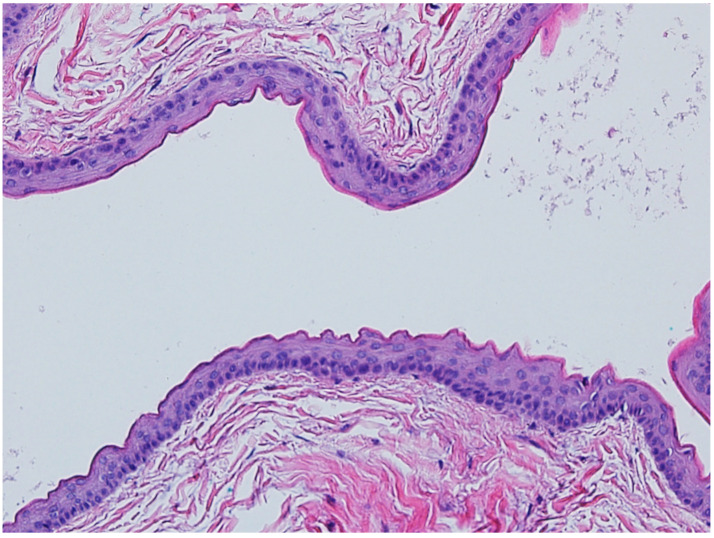
Steatocystomas are characterized as multiloculated cysts lined by stratified squamous epithelium with an undulating, eosinophilic cuticle on the inner cyst wall lining (hematoxylin and eosin, 20×).

Although cystic lesions are not considered neoplasms, steatocystomas have been included in this review because of their potential syndromic associations. These lesions may be seen in association with steatocystoma multiplex (OMIM #184500), an autosomal dominant disorder resulting from a mutation of *KRT17* on chromosome 17q21.2.1 [47,48]. Steatocystoma multiplex presents as an early onset of multiple translucent skin-colored to yellow dome-shaped papules or nodules on the trunk, head and neck, and limbs [49]. Histologically, steatocystomas are characterized as thin-walled dermal-based multiloculated cysts lined by stratified squamous epithelium lacking a granular layer and with an undulating, eosinophilic cuticle forming the inner cyst wall lining (Figure 8). Sebaceous lobules are usually identified within the cyst wall. Steatocystomas may become inflamed, infected, and scarred (steatocystoma multiplex suppurativum), causing cosmetic disfigurement [49]. Early recognition of a syndromic association in a patient with multiple steatocystomas, followed by appropriate counselling may alleviate some of the psychological issues associated with this syndrome [50].

Multiple steatocystomas may also be seen in pachyonychia congenita type 2 (OMIM #167210), which also results from mutations in keratin genes including *KRT17.* Affected patients may additionally present with severe nail abnormalities, painful palmoplantar keratoderma, oral leukokeratosis, vellus hair cysts, palmoplantar hyperhidrosis, natal or prenatal teeth, and follicular keratoses on the trunk and limbs [51].

## 4. Sweat Gland Tumors

### 4.1. Cylindroma/Spiradenoma

Cylindromas and spiradenomas are benign adnexal neoplasms most commonly affecting the scalp, face, and neck. Clinically, they present as firm, pink to reddish dome-shaped nodules. Cylindromas may coalesce into large masses on the scalp known as “turban tumors”, while spiradenomas are often painful and more likely to occur on the trunk or extremities. Lesions usually appear in adolescence or early adulthood and progressively increase in number and size [52,53].

**Figure 9:**
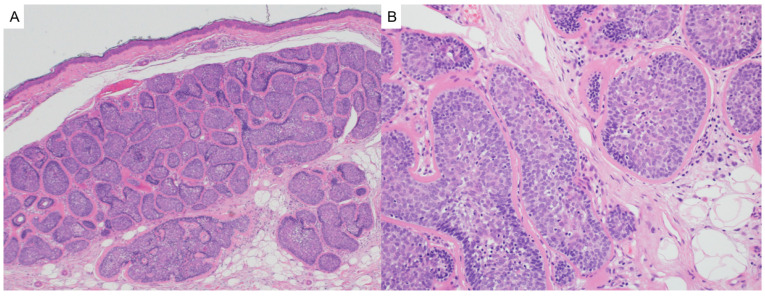
(**A**) Cylindromas show a jigsaw puzzle-like arrangement of nodules of basaloid cells containing or surrounded by eosinophilic basement membrane material (hematoxylin and eosin, 4×). (**B**) Peripheral cells can show palisading and are usually darker than the central cells of the nodules. There may be intraepithelial lymphocytes (hematoxylin and eosin, 20×).

**Figure 10:**
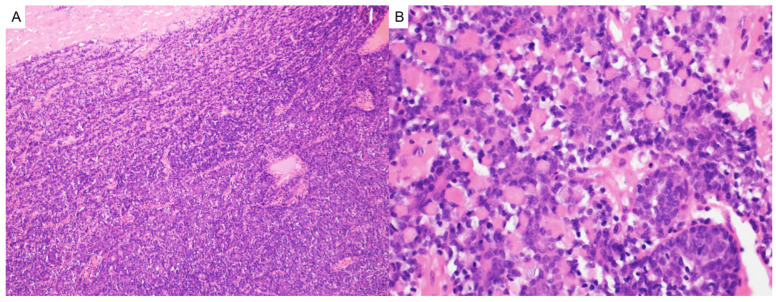
(**A**) Spiradenomas can show large nodules composed of diffuse sheets (hematoxylin and eosin, 10×) of (**B**) small basaloid cells intermixed with paler cells and lymphocytes with associated basement membrane material (hematoxylin and eosin, 40×).

Histologically, cylindromas demonstrate basaloid cells arranged in a distinctive “jigsaw puzzle” pattern surrounded by thick PAS-positive eosinophilic basement membrane material (Figure 9) [53,54]. Spiradenomas, in contrast, show lobular architecture with dual populations of small dark peripheral and large pale central cells, surrounded by prominent lymphocytic infiltrates (Figure 10) [52]. Hybrid tumors, called spiradenocylindromas, containing features of both may also occur.

Immunohistochemically, both tumors exhibit cytokeratin and SOX10 positivity [55], with myoepithelial markers such as calponin, SMA, S100, and p63 expressed in surrounding cells. Ductal differentiation is highlighted by CEA and EMA. Spiradenomas uniquely contain CD3+ T-cells and CD1a+ Langerhans cells [56]. Malignant transformation (to spiradenocarcinoma or cylindrocarcinoma) is rare but marked by an elevated Ki67 proliferative index and loss of characteristic MYB expression in spiradenomas [56,57].

These tumors are central to the diagnosis of Brooke–Spiegler syndrome (OMIM# 605041), an autosomal dominant condition caused by inactivating mutations in the tumor suppressor gene *CYLD*, located on chromosome 16q12-13. Brooke–Spiegler syndrome presents with multiple cylindromas, spiradenomas, spiradenocylindromas, and trichoepitheliomas and occasionally basal cell carcinomas or salivary gland tumors. It exhibits variable penetrance and significant phenotypic heterogeneity even within the same family [58]. Multiple cylindromas can also be found in familial cylindromatosis (OMIM #132700). Brooke–Spiegler syndrome and familial cylindromatosis are phenotypic variants of *CYLD* cutaneous syndrome.

Early identification of multiple adnexal tumors, especially in young individuals or those with a family history, should prompt evaluation for *CYLD* cutaneous syndrome. Genetic confirmation via *CYLD* mutation analysis is diagnostic. Regular follow-up is essential to monitor for malignant transformation and associated internal neoplasms [56].

### 4.2. Syringoma

**Figure 11:**
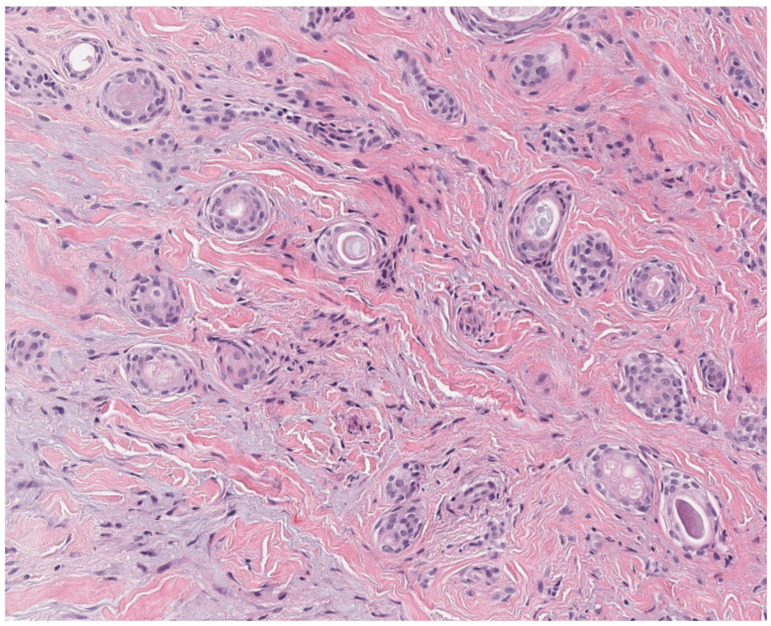
Syringomas show well-circumscribed superficial dermal proliferations of small nests, cords, and tadpole-like ducts composed of bland pale eosinophilic to clear cells (hematoxylin and eosin, 10×).

Syringomas are common tumors that typically present as small, skin-colored to yellow papules on the lower eyelids and periorbital areas. Eruptive syringomas present as multiple widespread scattered papules on the trunk and limbs, which may coalesce to form plaques [59,60]. Histologically, they are characterized as well-circumscribed proliferations of small ducts, nests, and cords of bland pale eosinophilic to clear cells in the superficial dermis (Figure 11). Ducts can have a tadpole-like appearance with comma-like tails. While syringomas usually occur sporadically, they have also been found to be associated with Down syndrome (OMIM #190685, characterised by trisomy 21) and rarely in other syndromes, such as Nicolau Balus syndrome, Brooke–Spiegler syndrome (OMIM# 605041), Costello syndrome (OMIM# 218040), and steatocystoma multiplex (OMIM #184500) [61,62,63,64,65]. In Down syndrome, syringomas are most commonly found around the eyes [66,67]. Other dermatologic manifestations that may be seen in Down syndrome include inflammatory dermatoses (such as atopic dermatitis), alopecia areata, and elastosis perforans serpiginosa [62]. Patients with Down syndrome have an increased risk of acute leukemias [68]. Nicolau Balus syndrome is a rare disorder characterized by syringomas, atrophorderma vermiculata, and milia [69].

## 5. Neural Tumors

### Neurofibromas

Neurofibromas are benign peripheral nerve sheath tumors composed of a mixture of Schwann cells, fibroblasts, mast cells, and perineurial cells. While they can arise sporadically, the presence of multiple neurofibromas is a cardinal feature of Neurofibromatosis Type 1 (NF1, OMIM# 162200), an autosomal dominant neurocutaneous syndrome caused by mutations in the *NF1* gene on chromosome 17q11.2. This gene encodes neurofibromin, a tumor suppressor that negatively regulates RAS signaling through its GTPase-activating protein function. Loss of neurofibromin leads to dysregulated cell proliferation, especially in neural crest–derived tissues [70,71].

**Figure 12:**
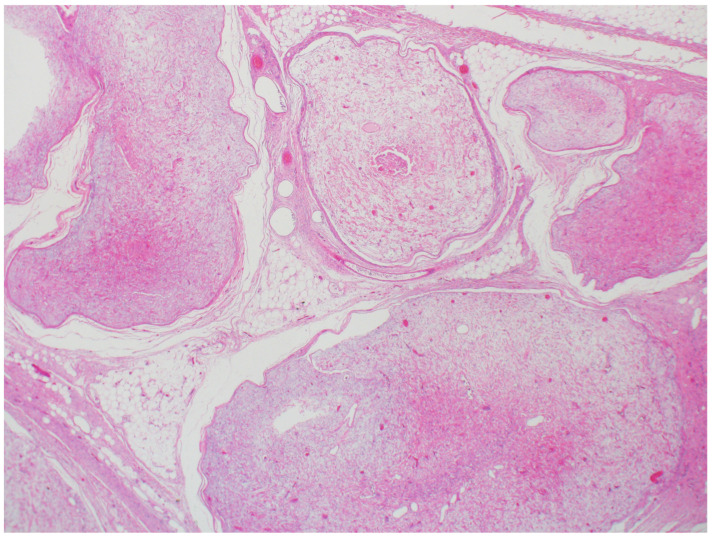
Plexiform neurofibroma shows an irregular, multinodular growth of expanded nerves (hematoxylin and eosin, 2×).

Clinically, neurofibromas typically begin to appear during adolescence and increase in number and size with age. Cutaneous neurofibromas are soft, skin-colored to violaceous papules or nodules with a characteristic “buttonhole” invagination upon palpation. Plexiform neurofibromas, which involve multiple nerve fascicles in a tortuous, infiltrative manner, are often congenital and considered pathognomonic for NF1 [72]. These lesions carry a 10% lifetime risk of malignant transformation into malignant peripheral nerve sheath tumors (MPNST), particularly when rapidly enlarging or associated with pain or neurologic changes. Histologically, neurofibromas are unencapsulated and composed of S100-positive Schwann cells and CD34-positive fibroblasts within a myxoid or collagenous stroma. Immunohistochemistry may also demonstrate CD117-positive mast cells and EMA-positive perineurial cells [73]. Plexiform subtypes show a more diffuse, infiltrative growth along large nerve trunks and may involve adjacent soft tissue and skin (Figure 12).

NF1 is a multisystem disorder with complete penetrance and highly variable expressivity. The diagnostic criteria include two or more of the following: six or more café-au-lait macules (>5 mm in prepubertal or >15 mm in postpubertal individuals), axillary or inguinal freckling, two or more neurofibromas of any type or one plexiform neurofibroma, optic pathway glioma, two or more iris Lisch nodules (iris hamartomas), distinctive osseous lesions such as sphenoid wing dysplasia or tibial pseudoarthrosis, and a first-degree relative with NF1 [74]. Lisch nodules are seen in over 90% of adults with NF1 and are considered a sensitive ophthalmologic finding [73]. Additional systemic features may include learning disabilities, skeletal dysplasia, vascular abnormalities (e.g., renal artery stenosis, hypertension), and increased risk of malignancies including MPNSTs, breast cancer (especially in women under 50), and gastrointestinal stromal tumors [73,75].

Cutaneous neurofibromas, while benign, are a major cause of disfigurement and quality-of-life burden in patients with NF1. Their number increases throughout life and may surge during puberty or pregnancy due to hormonal influences [76]. Ongoing surveillance and multidisciplinary management, including regular dermatologic, ophthalmologic, neurologic, and genetic evaluations, are essential for early detection and management of complications in this complex, lifelong condition. The presence of multiple neurofibromas or a plexiform neurofibromas should prompt consideration of NF1.

## 6. Smooth Muscle Tumors

### Leiomyomas

**Figure 13:**
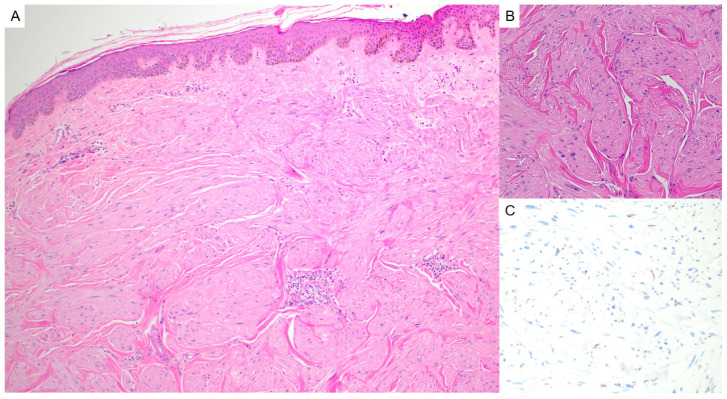
(**A**) Leiomyomas are composed of interlacing bundles of spindle cells (hematoxylin and eosin, 10×) with (**B**) eosinophilic cytoplasm and blunt-ended nuclei (hematoxylin and eosin, 20×). (**C**) Reed syndrome associated leiomyomas demonstrate loss of cytoplasmic expression with FH immunohistochemistry (FH, 20×).

Leiomyomas are benign smooth muscle tumors that may occur sporadically or in association with genetic syndromes [77]. Clinically, cutaneous leiomyomas present as firm, skin-colored to erythematous papules or nodules, often painful to touch or with cold exposure [78]. Histologically, these tumors are composed of interlacing bundles of spindle cells with eosinophilic cytoplasm and blunt-ended nuclei, consistent with smooth muscle differentiation (Figure 13) [79]. Immunohistochemically, they are positive for smooth muscle markers such as desmin, caldesmon and smooth muscle actin (SMA) [80].

In the syndromic context, multiple cutaneous and uterine leiomyomas may indicate Hereditary Leiomyomatosis and Renal Cell Cancer (HLRCC, OMIM #150800), also known as Reed syndrome. This autosomal dominant condition is caused by germline mutations in the *FH* gene, located on chromosome 1q43, which encodes fumarate hydratase (FH), a key enzyme in the Krebs cycle. Loss of FH function leads to metabolic dysregulation and increased risk for malignancy, particularly type 2 papillary renal cell carcinoma [81]. Immunohistochemical staining for FH can demonstrate loss of cytoplasmic expression in lesional cells, supporting a syndromic diagnosis. Additionally, affected tumors may show positive staining for 2-succinocysteine (2SC), a surrogate marker for FH deficiency [82].

Given the potential for life-threatening, aggressive renal cancer, recognition of multiple cutaneous leiomyomas with loss of FH expression and a history of uterine leiomyomas should prompt evaluation for HLRCC, including genetic counseling, imaging surveillance, and screening of at-risk family members [83].

## 7. Vascular Tumors

### 7.1. Angiokeratoma Corporis Diffusum

**Figure 14:**
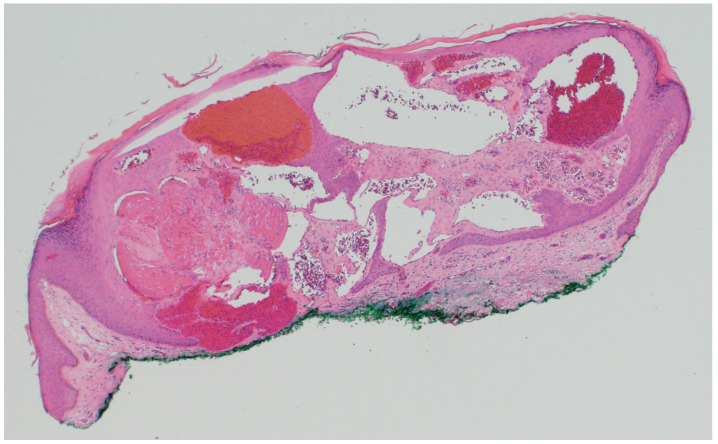
Angiokeratomas are composed of dilated blood vessels in the papillary dermis the appear to herniate into the overlying hyperplastic epidermis (hematoxylin and eosin, 4×).

Angiokeratoma corporis diffusum is a vascular lesion that presents in childhood as clusters of dark red to black papules, typically distributed over the lower trunk, buttocks, and thighs. These lesions represent dilated capillaries in the superficial dermis with overlying epidermal hyperkeratosis. Histologically, they reveal telangiectatic blood vessels in the papillary dermis, and can be accompanied by epidermal acanthosis and hyperkeratosis (Figure 14). Angiokeratoma corporis diffusum may demonstrate PAS-positive and Sudan black-positive lipid granules within endothelial cells, pericytes, and fibroblasts on frozen section examination.

Angiokeratoma corporis diffusum is a hallmark of Anderson–Fabry disease (OMIM #301500), a rare X-linked recessive lysosomal storage disorder caused by deficiency of the enzyme α-galactosidase A. The *GLA* gene, located on chromosome Xq22, is responsible for encoding this enzyme, and its deficiency leads to systemic accumulation of globotriaosylceramide in various tissues. Ultrastructural examination of tissue by electron microscopy, particularly kidney biopsies, reveals the pathognomonic presence of lamellar cytoplasmic inclusions, often described as “zebra bodies” [84,85].

In addition to cutaneous findings, Anderson–Fabry disease is associated with a range of systemic features, including renal failure, cardiomyopathy, acroparesthesias, and cerebrovascular disease. Rarely, angiokeratoma corporis diffusum may occur with deficiencies of other lysosomal enzymes such as α-L-fucosidase (fucosidosis, OMIM #230000) or β-mannosidase (β-mannosidosis, OMIM #248510), but Anderson–Fabry disease remains the most common association [86,87]. Recognition of a syndromic association and early diagnosis is critical, as enzyme replacement therapy may slow disease progression and improve quality of life [88].

### 7.2. Other Vascular Anomalies

The International Society for the Study of Vascular Anomalies classification system highlights an exhaustive list of other vascular tumors and malformations with syndromic associations [89]. These include vascular anomalies associated with Sturge–Weber syndrome (OMIM# 185300), Capillary Malformation-Arteriovenous Malformation syndromes (OMIM# 608354 and 618196), Hereditary Hemorrhagic Telangiectasia syndrome (OMIM# 175050), Klippel–Trenaunay syndrome (OMIM# 149000), and Proteus syndrome (OMIM# 158350) [89]. Further details on these vascular lesions and syndromes are outside the scope of this review.

## 8. Adipocytic Tumors

### Lipomas

**Figure 15:**
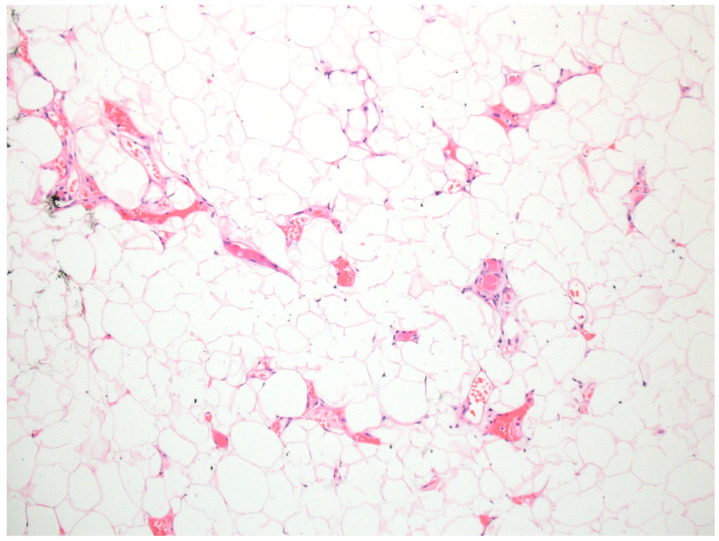
Lipomas are composed of mature adipose tissue with angiolipomas demonstrating a vascular component composed of clusters of small vessels with fibrin thrombi (hematoxylin and eosin, 10×).

Lipomas are benign tumors composed of mature adipocytes and are the most common soft tissue neoplasms. Clinically, they appear as soft, mobile, subcutaneous nodules that are typically painless and slow-growing. Histological examination demonstrates mature adipose tissue (Figure 15). Multiple lipomas may arise sporadically or suggest an underlying genetic syndrome.

Several syndromes are associated with multiple lipomas, including Cowden syndrome (OMIM #158350), Proteus syndrome (OMIM #176920), and familial multiple lipomatosis (OMIM #151900). Cowden syndrome is an autosomal dominant disorder caused by mutations in the *PTEN* gene, located on chromosome 10q23.31, and is part of the PTEN hamartoma tumor syndrome spectrum. Affected individuals may have lipomas alongside trichilemmomas, oral papillomas, and an increased risk of breast, thyroid, and endometrial cancers [90]. Proteus syndrome is a rare, mosaic condition caused by somatic activating mutations in the *AKT1* gene, located on chromosome 14q32.33. It is characterized by asymmetric overgrowth of bones, skin, and other tissues, including lipomas, connective tissue nevi, and vascular malformations [91]. Familial multiple lipomatosis is an autosomal dominant condition associated with variants in exon 5 of the *HMGA2* gene, located on chromosome 12q14-15 [92]. It is characterized by the presence of multiple, slow-growing lipomas and angiolipomas on the trunk and limbs and increased body habitus.

Recognition of multiple lipomas in the context of the syndromic features mentioned should prompt genetic evaluation and multidisciplinary management.

## 9. Fibrohistiocytic/Fibrovascular Tumors

### 9.1. Superficial Angiomyxoma

**Figure 16:**
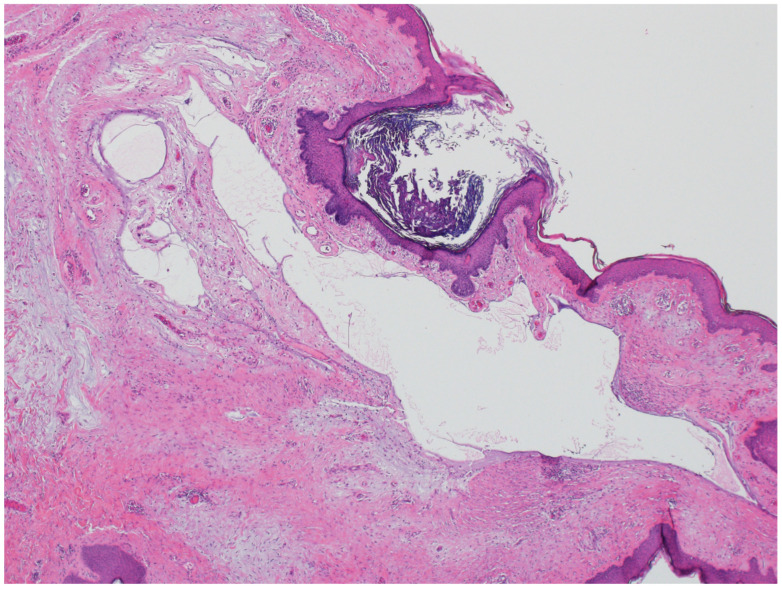
Superficial angiomyxomas demonstrate a well-circumscribed, unencapsulated proliferation of spindle cells in a prominent myxoid matrix with interspersed blood vessels and entrapped epithelial elements (hematoxylin and eosin, 4×).

Superficial angiomyxoma is a rare, benign cutaneous mesenchymal tumor. Clinically, these lesions present as polypoid or nodular masses, often on the trunk, head, or neck. Histologically, they are characterized by a well-circumscribed but non-encapsulated proliferation of spindle or stellate cells in a prominent myxoid matrix, interspersed with small, thin-walled blood vessels, neutrophils, and occasional entrapped benign epithelial elements (Figure 16). Immunohistochemistry typically shows positivity for CD34 [93].

Superficial angiomyxomas can occur sporadically or in association with Carney complex (OMIM #160980), an autosomal dominant multiple neoplasia syndrome. Carney complex is caused by mutations in the *PRKAR1A* gene, which encodes a regulatory subunit of protein kinase A and is located on chromosome 17q24. In this context, superficial angiomyxomas may be multiple and recurrent. Carney complex is also associated with cardiac myxomas, spotty skin pigmentation (lentigines), endocrine tumors (e.g., pituitary adenomas, adrenal tumors), and other myxoid neoplasms [94,95,96]. Recognition of this tumor, especially in younger individuals or in the presence of other stigmata, should prompt consideration of syndromic evaluation and genetic counselling [83].

### 9.2. Facial Angiofibromas/Acral Fibrokeratomas

Facial angiofibromas (adenoma sebaceum) and acral fibrokeratomas can be associated with tuberous sclerosis complex (TSC), a multisystem genetic disorder. Angiofibromas associated with TSC typically manifest as multiple small, reddish papules distributed symmetrically across the central face, particularly the nasolabial folds and cheeks [97,98]. Acral fibrokeratomas, or “Koenen tumors”, are periungual or subungual fibromas that emerge in adolescence or adulthood and may be painful or disfiguring [99].

**Figure 17:**
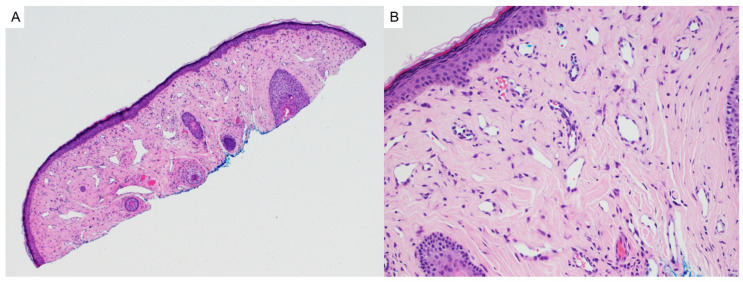
(**A**) Angiofibroma is a somewhat polypoid dermal lesion (hematoxylin and eosin, 2×) (**B**) composed of bland dermal fibroblasts and collagen bundles with prominent vasculature (hematoxylin and eosin, 10×).

**Figure 18:**
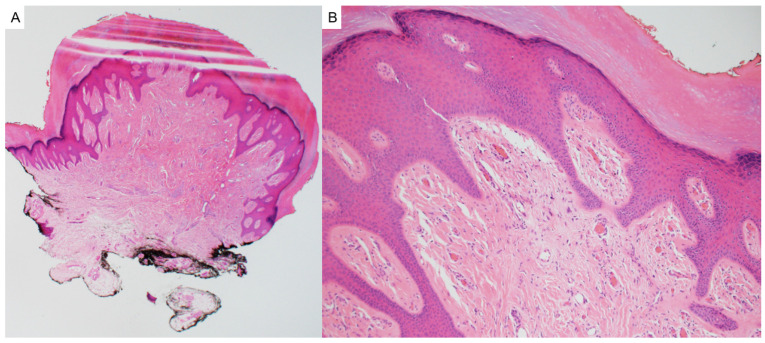
(**A**) Acral fibrokeratoma is a polypoid dermal lesion (hematoxylin and eosin, 2×) (**B**) demonstrating hyperkeratosis, acanthosis, and a bland dermal proliferation of fibroblasts, collagen bundles, and prominent blood vessels (hematoxylin and eosin, 10×).

Histologically, angiofibromas lesions demonstrate scattered bland dermal fibroblasts and collagen bundles with prominent vasculature (Figure 17). Acral fibrokeratomas demonstrate a polypoid lesion with hyperkeratosis, acanthosis, dermal fibroblasts, thick collagen bundles, and scattered blood vessels (Figure 18). Immunohistochemistry findings are generally nonspecific but may aid in ruling out mimics [100].

Tuberous sclerosis complex is caused by mutations in either the *TSC1* (hamartin, located on chromosome 9q34) (OMIM #191100) or *TSC2* (tuberin, located on chromosome 16p13.3) (OMIM #613254) genes and follows an autosomal dominant inheritance pattern with variable expressivity. The syndrome is associated with a wide spectrum of manifestations, including cortical tubers, subependymal giant cell astrocytomas, renal angiomyolipomas, cardiac rhabdomyomas, and pulmonary lymphangioleiomyomatosis [101]. Multiple clustered angiofibromas and/or acral fibrokeratomas, especially in young patients, should prompt consideration of these syndromes.

## 10. Melanocytic Tumors

### 10.1. BAPomas

**Figure 19:**
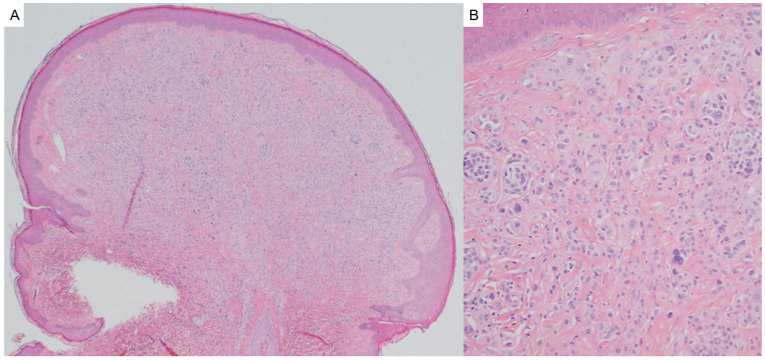
(**A**) BAP-1 inactivated melanocytic tumors can show a polypoid appearance (hematoxylin and eosin, 2×) and (**B**) are composed of a predominantly intradermal proliferation of epithelioid melanocytes with abundant cytoplasm, well-defined cellular borders, vesicular nuclei, distinct nucleoli, and occasional multinucleation (hematoxylin and eosin, 10×).

BAP-1 inactivated melanocytic tumors (BAPomas) are generally indolent melanocytic tumors associated with inactivating mutations in the *BAP1* gene (BRCA1-associated protein-1), a tumor suppressor gene on chromosome 3p21.1 [102]. Clinically, BAPomas usually present as dome-shaped, skin-colored to lightly pigmented papules that may resemble banal nevi and may arise anywhere on the body. Histologically, BAPomas are distinguished by a predominantly intradermal proliferation of epithelioid melanocytes with abundant cytoplasm, well-defined cellular borders, vesicular nuclei, distinct nucleoli, and occasional multinucleation (Figure 19) [103,104]. They may arise in association with a conventional nevus. Loss of nuclear BAP1 expression on immunohistochemistry is helpful in confirming a diagnosis of BAPoma [105].

BAPomas may occur sporadically or may arise in association with BAP1 tumor predisposition syndrome (BAP1-TPDS, OMIM #614327). BAP1-TPDS is an autosomal dominant disorder caused by germline BAP1 mutations [102]. BAPomas are frequently multiple and appear in early adulthood in BAP1-TPDS. BAP1-TPDS is associated with an increased risk for uveal melanoma, mesothelioma, cutaneous melanoma, and renal cell carcinoma [106]. Identification of multiple BAPomas in a patient with a personal or family history suggestive of BAP1-TPDS should prompt genetic testing and surveillance for associated malignancies.

### 10.2. Pigmented Epithelioid Melanocytoma

**Figure 20:**
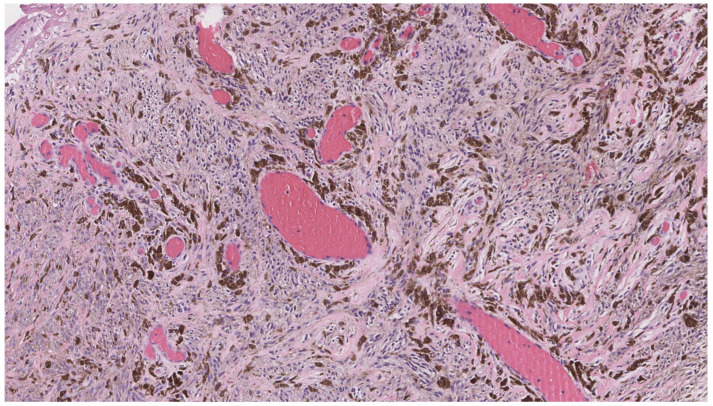
Pigmented epithelioid melanocytoma demonstrates heavily pigmented epithelioid and spindle-shaped melanocytes arranged in nests or sheets within the dermis (hematoxylin and eosin, 10×).

Pigmented epithelioid melanocytoma (PEM), also known as epithelioid blue nevus-like tumor, is a rare, deeply pigmented melanocytic neoplasm characterized by low to intermediate malignant potential and frequent sentinel lymph node involvement, though distant metastasis is rare [107,108]. Clinically, it presents as a darkly pigmented nodule, typically located on the extremities or trunk [108,109]. Histologically, PEM is composed of heavily pigmented epithelioid and spindle-shaped melanocytes arranged in nests or sheets within the dermis (Figure 20). Immunohistochemistry demonstrating loss of PRKAR1A expression can be useful for confirming a diagnosis of PEM [110].

PEM can occur sporadically or be associated with Carney complex (OMIM #160980), a multiple neoplasia syndrome caused by mutations in the *PRKAR1A* gene on chromosome 17q24 [111]. In Carney complex, PEMs may occur alongside cardiac and cutaneous myxomas, endocrine tumors, schwannomas, and lentigines. Carney complex is inherited in an autosomal dominant fashion [112]. The presence of multiple PEMs should prompt consideration for a syndromic association.

### 10.3. Dysplastic/Atypical Nevi

Dysplastic/atypical nevi, formerly known as “Clark’s nevi”, demonstrate clinical atypia compared to ordinary nevi, including larger size, irregular borders, and color variation [113]. On histology, they are characterized by the presence of architectural disorder (shoulder phenomenon, bridging of junctional nests between elongated rete ridges, pagetoid scatter, and lentiginous growth), cytologic atypia (enlarged and pleomorphic nuclei, hyperchromasia, and prominent nucleoli), and host response (lamellar and concentric fibroplasia of the papillary dermis and a patchy superficial lymphocytic infiltrate) [114]. Multiple dysplastic/atypical nevi may be associated with Familial Atypical Multiple Mole and Melanoma Syndrome (OMIM# 155600), a typically autosomal dominant disorder caused by inherited mutations in tumor suppressor genes associated with melanoma susceptibility, such as *CDKN2A*, *CDK4*, and *ARF* [115]. Individuals with these syndromes have a significantly increased risk for the development of melanoma and an increased risk for the development of internal malignancies, especially pancreatic carcinoma [115,116,117]. The presence of multiple dysplastic/atypical nevi in an individual should prompt regular dermatologic surveillance for the early detection and management of melanoma in these patients and their families and consideration for genetic testing.

## 11. Conclusions

Benign skin tumors with syndromic associations represent a valuable and often underrecognized opportunity for the early identification of genetic syndromes. Though often overlooked due to their innocuous appearance, these lesions can display subtle, yet diagnostically meaningful features on histopathologic evaluation, particularly when interpreted with clinical context [118]. When such findings present in multiples, demonstrate distinct morphological features, or occur in younger individuals or with a relevant family history, a syndromic etiology should be considered [2]. Timely recognition in these cases is critical, as it allows for risk stratification and surveillance for associated internal malignancies [3].

This review synthesizes the clinical and histological features of benign cutaneous neoplasms with known syndromic associations (Table 1). In this diagnostic process, dermatopathologists play a pivotal role. Their careful evaluation, supported by ancillary testing, is often essential for uncovering the systemic implications of these lesions even in the absence of overt clinical signs [119]. Once a syndromic diagnosis is established, patient management should include regular surveillance for associated malignancies and referral for genetic counselling for both the patient and at-risk family members [2]. Notably, in several syndromes, including Muir–Torre, Cowden, and Birt–Hogg–Dubé, the skin may be the earliest or only organ involved at presentation. Failure to recognize these early cutaneous signs within their syndromic context may delay critical interventions and allow for progression of occult malignancies [120,121]. It is thus paramount that dermatopathologists pay attention to clinical and histological clues in these lesions that may suggest a syndrome and guide the referring clinician to consider further screening and genetic counselling for these patients and their families.

## Figures and Tables

**Table 1:** Benign cutaneous neoplasms with syndromic associations and the genetic, dermatologic, and oncologic features associated with these syndromes.

Entity	Associated Syndrome(s)	OMIM #	Syndrome Associated Gene(s)	Other Syndrome Associated Skin Lesions	Syndrome Associated Malignancy
Fibrofolliculoma/Trichodiscoma	Birt–Hogg–Dubé syndrome	135150	*FLCN*	Facial angiofibromas, acrochordons	Renal cell carcinoma
Tricholemmoma	PTEN Hamartoma Tumor Syndrome (e.g., Cowden, Bannayan–Riley–Ruvalcaba, Proteus)	153480 (Cowden) 158350 (Bannayan–Riley–Ruvalcaba), 176920 (Proteus)	*PTEN*	Acral keratoses, lipomas, milia, mucocutaneous papillomas	Breast, thyroid, endometrial cancers
Pilomatricoma	Gardner’s Syndrome	175100	*APC*	Epidermoid cysts, fibromas	Colon cancer
Basaloid follicular hamartoma	Nevoid basal cell carcinoma syndrome/Gorlin syndrome	109400	*PTCH1*, *SUFU*	Basal cell carcinoma, palmar or plantar pits	Medulloblastoma
Sebaceous adenoma/epithelioma	Muir–Torre syndrome	158320	*MSH2*, *MLH1*, *MSH6*, *PMS2*	Sebaceous carcinoma, keratoacanthoma	Colorectal, genitourinary, endometrial, gastric, pancreatic, and breast carcinomas
Steatocystoma	Steatocystoma multiplex and pachyonychia congenita type 2	184500 (steatocystoma multiplex), 167210 (pachyonychia congenita type 2)	*KRT17*	Severe nail abnormalities, painful palmoplantar keratoderma, vellus hair cysts, follicular keratoses in pachyonychia congenita type 2	-
Cylindroma/Spiradenoma/Spiradenocylindroma/Trichoepitheliomas	*CYLD* cutaneous syndrome, including: Brooke–Spiegler syndrome, familial cylindromatosis, multiple familial trichoepitheliomas	605041 (Brooke–Spiegler syndrome) 132700 (familial cylindromatosis) 601606 (multiple familial trichoepitheliomas)	*CYLD*	Basal cell carcinomas and malignant neoplasms arising from spiradenoma, cylindroma, or spiradenocylindroma	Salivary gland tumors
Syringoma	Down syndrome, rare others	190685	Trisomy 21	Inflammatory dermatoses (Down syndrome)	Leukemia (Down syndrome)
Neurofibroma	Neurofibromatosis Type 1	162200	*NF1*	Café-au-lait macules, axillary/inguinal freckling, malignant peripheral nerve sheath tumor	Malignant peripheral nerve sheath tumor, breast cancer
Leiomyoma	Hereditary leiomyomatosis and renal cell cancer (Reed syndrome)	605839	*FH*	-	Type 2 papillary renal cell carcinoma
Angiokeratoma corporis diffusum	Anderson–Fabry disease	301500	*GLA* (α-galactosidase A)	-	-
Lipoma	Cowden and Proteus syndromes	153480 (Cowden), 176920 (Proteus)	*PTEN* (Cowden), *AKT1* (Proteus)	-	Breast, thyroid, endometrial cancers (Cowden)
Superficial angiomyxoma	Carney complex	160980	*PRKAR1A*	Spotty skin pigmentation	Melanocytic tumors, endocrine neoplasms
Facial angiofibromas/Acral fibrokeratomas	Tuberous sclerosis complex	191100 (TSC1), 613254 (TSC2)	*TSC1*, *TSC2*	-	-
BAPoma	BAP1 tumor predisposition syndrome	614327	*BAP1*	Cutaneous melanoma	Uveal melanoma, mesothelioma, renal cell carcinoma
Pigmented epithelioid melanocytoma	Carney complex (in some cases)	160980	*PRKAR1A*	Spotty skin pigmentation	Melanocytic tumors, endocrine neoplasms
Dysplastic/atypical melanocytic nevi	Familial Atypical Multiple Mole and Melanoma Syndrome	155600	*CDKN2A*, *CDK4*, *ARF*	Melanoma	Internal malignancies, especially pancreatic carcinoma
